# Empowering minds and fostering inclusion: ELT graduate students’ experiences with critical pedagogy

**DOI:** 10.3389/fpsyg.2025.1678565

**Published:** 2025-11-18

**Authors:** Senem Zaimoğlu, Aysun Dağtaş

**Affiliations:** Department of Translation and Interpreting, Çağ University, Mersin, Türkiye

**Keywords:** critical pedagogy, transformative learning, ELT teacher education, reflective practice, disorienting dilemmas

## Abstract

**Introduction:**

This research focuses on how graduate students in a Master’s level English Language Teaching (ELT) program in Türkiye experienced transformative learning through a course on critical pedagogy (CP). Grounded in Mezirow’s transformative learning theory and Freirean principles, the study explores how engagement with the sociopolitical dimensions of language education challenged participants’ assumptions and transformed their cognitive, emotional, and relational understandings of teaching.

**Methods:**

Data were collected from eight participants through weekly reflective journals structured around Gibbs’ Reflective Cycle that guides individuals to reflect systematically on their experiences and follow-up semistructured interviews. Following Braun and Clarke’s six-phase framework to thematic analysis, five overarching themes were identified that encapsulate the participants’ experiences: Confronting Disorienting Dilemmas, Shifting Perspectives (Cognitive Transformations), Navigating Emotional Journeys, Evolving Classroom Relationships, and Embracing Transformative Learning Processes.

**Results:**

The results revealed that participants encountered disorienting dilemmas that prompted self-examination and shifts in identity, beliefs, and pedagogical stances. While students valued dialogic learning, critical inquiry, and real-world connections, some reported discomfort when addressing controversial topics—highlighting persistent hierarchical dynamics within educational settings.

**Discussion:**

Despite these tensions, participants demonstrated growing commitment to inclusive and socially engaged teaching. This research contributes to inclusive teacher education by demonstrating how structured reflection and emotionally responsive learning environments can foster transformation. It also calls for further research on the long-term enactment of critical pedagogy in diverse institutional contexts.

## Introduction

The concept of empowerment and inclusion has become an important aspect of contemporary language education, particularly in ELT as this field is shaped by power, ideology, and global inequalities. In this regard, CP, with its foundations in the work of Freire (1970), posits that education should serve as a transformative force for social change, addressing oppression, inequality, and human suffering rather than merely reproducing the existing social order. Accordingly, rather than framing the learner as a passive recipient of knowledge as in Freire’s “banking model,” —where teachers deposit information into students who merely memorize and store it without questioning—CP positions students as active agents who interrogate societal contradictions and pursue transformative praxis through reflection and action. In doing so, CP provides opportunities for students to challenge assumptions that are regarded as factual, and to reimagine themselves in the world through and with language, thereby leading to epistemological and ontological change. Kincheloe (2008) argues that this disruption is essential for education to function as a vehicle for a more ‘just and humane’ world. Consistent with this orientation, CP challenges students to question received knowledge through problem-posing techniques that disrupt the traditional flow of instruction. It encourages them to be creators and not just consumers of knowledge (Jeyaraj and Harland, 2014). Taken together, in these contexts, transformations take place as old assumptions, values, and feelings give way to new ways of knowing, seeing, and being in the world.

Relatedly, the fundamental tenets of CP resonate with many scholars across several areas of education, including ELT, in which language(s) are increasingly viewed not only as the medium for communication but also as a carrier of cultural capital, power, and identity (e.g., Norton and Toohey, 2004). In this sense, language learning is not a neutral act but is embedded in ideologies acting upon the learners’ access, their agency, and their sense of belonging to a community (Pennycook, 2001).

Against this broader backdrop, the transformation in teacher identity and classroom practice is often gradual and non-linear, yet it can lead to more engaged, inclusive, and critically conscious practitioners. Consequently, given the limited empirical research, especially in Türkiye, on the experiences of ELT graduate students who learn about and teach with CP, there is an urgent need to examine how ELT graduate students experience CP in real classroom settings: What challenges do they face? How do they navigate deliberately uncomfortable situations or disorienting dilemmas? In what ways do they resist, adapt or incorporate CP principles? As such, asking these questions is necessary, not only to understand how CP has shaped teacher identity and teacher practice, but also to be able to create more effective and inclusive teacher education programs.

Accordingly, by prioritizing student voice and lived experience, this study investigates how ELT graduate students experience, interpret and negotiate the principles of CP in their courses and in their teaching practice. In doing so, it contributes to the growing—yet still underdeveloped—literature on CP in Turkish ELT and offers practical insights for educators and policy-makers committed to fostering more inclusive, equitable, and transformative language teacher education through the lens of students’ lived experiences and reflective engagement.

## Literature review

### CP in ELT: marginalization and the empirical gap

Although critical pedagogy (CP) is becoming more often referred to as a global phenomenon in education, it is unevenly regulated within mainstream ELT (Sanjakdar and Apple, 2024). Within a Turkish context, many ELT programs still situate themselves in technocratic and depoliticized frames overall, taking priority over the situational, social, political, cultural, and historical contexts of language (Ordem, 2022). As a result, student-teachers may be less likely to feel prepared to counter dominant ideologies or to foster inclusive and equitable classrooms. Consistent with previous findings that critical work often lives on the edges of mainstream exam-driven curricula—even when TE introduces critiques of the material and CDA/CLA (Wallace, 1999, 2003; Cots, 2006; Martínez, 2012), recent work across global contexts suggests it can happen, but again, with limits. In China, there is CLA-oriented pedagogy work and SFL-based critical-literacy tasks using news texts that have provided measurable learning benefits and tangible classroom designs (Huang and Guo, 2024; Luo and Xie, 2025). In Indonesia, higher-education classes that enact the core CP pedagogical frames (e.g., dialogue, problem-posing, praxis) report language and social benefits, as well as limits with participation and alignment (Yulianto et al., 2024). In the MENA context, in Egypt, university EFL teachers describe some early and uneven critical-literacy work influenced by institutional pressures (Abdel Latif, 2022). In Japan, recent research demonstrates how critical language pedagogy at the university level is responsive to local realities and student reception (Jackson and Kennett, 2025). Overall, these studies indicate that CP/CLA/CDA is gaining momentum globally; however uptake is steered by assessment regimes, materials, and teacher preparation, rather than just not being present.

This inequity is not simply a function of what is selected to study; it also creates a pedagogical disposition. If pacing is grounded in coverage and assessment alignment, classroom talk typically values accuracy and efficiency to a higher degree than inquiry and dialogue—conditions in which “safe” topics and closed tasks flourish, while dialogic practices of problem posing, perspective taking, and critical analysis of texts to the fore (Goodspeed and Ruf, 2025; Luo and Xie, 2025). At a programmatic level, mentoring/practicum feedback can promote compliance with procedures over open refusals for risk-taking in reflection, implicitly signaling to novices that critical engagement sits at the margins. As such, many student-teachers find themselves in a double bind, they endorse the ethical and educational value of CP but perceive few legitimate opportunities to enact it within high-stakes, exam-driven ecologies (Goodspeed and Ruf, 2025).

Cross-context reviews similarly reveal that critical frameworks can be used in EFL settings, but they are refracted through local ideologies, assessment cultures, and materials policies. Recent research in adult ELT and in critical pedagogy scholarship highlighted both expansion and constraint, adding to foundational understandings (Norton and Toohey, 2004; Canagarajah, 2005) with new mappings (Askari and Behdarvandirad, 2025; Paesani and Menke, 2025). In the Gulf, larger scale research in Saudi EFL points to how textbook design and policy mediate what counts as legitimate classroom work, a lever that interacts with attempts to foreground critical reading (Alshumaimeri and Alharbi, 2024). In Türkiye specifically, a scoping review – covering 2015-May 2022 – mapped 34 publications and identified takes in CP research as recurring strands (teacher/learner perspectives; curriculum critique; materials/methods analysis; course design) as a way to signify both growth and known gaps in CP research – including the need for more empirical studies with pre-service teachers to examine their experiences with CP in courses and practicums (Kızıldağ, 2023).

It is clear that beyond Türkiye, CP/critical literacy has been put into practice in a number of ELT teacher-education and classroom contexts (e.g., via materials critique as well as CDA/CLA tasks); as well as in larger (non-ELT) teacher-education programs focused on dialogic reflections and “rights” analyses of classroom policy (Benesch, 2001/2017). These implementation studies demonstrate that CP is not just an aspiration but may be taught in concrete terms if curricula create transparency around critical language work and if assessment criteria are made clear.

### How change happens: transformative learning within critical pedagogy

In contexts where CP principles are introduced, the process of internalizing and enacting them is rarely straightforward. Transformative learning, as theorized by Mezirow (1997), involves a fundamental shift in one’s perspective, often triggered by a “disorienting dilemma” that disrupts prior assumptions and leads to deep critical reflection. This process allows learners to revise their habitual ways of thinking and become more open, reflective, and inclusive in their beliefs and actions (Cranton, 2011). Within the framework of CP, this transformation extends beyond cognitive restructuring; it also encompasses action, as learners are called to engage with the world and challenge injustice (Wink, 2000). Transformation is thus both an internal and external process—shaping identity, social consciousness, and agency (Kincheloe, 2008). In ELT teacher education, this shift can be especially powerful, prompting future teachers to question traditional hierarchies, confront discomfort, and adopt pedagogical practices rooted in equity and inclusion. Yet the process is rarely linear; it is influenced by emotional resistance, classroom dynamics, and institutional norms. Nevertheless, when supported by reflective dialogue, experiential learning, and real-world problem-posing, transformative learning can foster both personal growth and professional empowerment.

To make sense of these processes, in teacher education programs, the intersection of Freirean critical pedagogy and Mezirow’s transformative learning theory offers a rich lens for analyzing how student-teachers make sense of their learning experiences. While Freire provides the ethical and political foundations of CP, emphasizing praxis, dialogue, and social transformation, Mezirow elucidates the internal cognitive and emotional journey of transformation that adult learners undergo when they critically reassess their beliefs. Together, these frameworks enable a holistic understanding of not only what is taught in a CP course but also how it is received, resisted, and (potentially) internalized by learners. Drawing from the literature on transformative learning theory, transformative learning views transformation as a “major change in perspective” that encourages people to think in more open, flexible, and well-reasoned ways (Cranton, 2011). However, in the context of CP, transformation must be viewed as more than simply an internal change of perspective, it involves enacting or “doing something” (Jeyaraj and Harland, 2014). CP often stands out from many contemporary pedagogical orientations because it advances the notion of doing something with knowledge, that knowledge gain can lead to social change (Wink, 2000). In this context, transformation is not limited to the classroom, but rather occurs in the broader community, where students can struggle against established societal structures and norms. For this transformation to be meaningful, theory must be attached and reattached to lived experience and allow academic work and social action to interdependently inform each other; like the two sides of a coin (Kincheloe, 2008).

Emotions also play a constitutive—not incidental—role in this trajectory. Confusion, anxiety, or discomfort often signal that a meaning perspective is being challenged; equally, relief, clarity, or resolve can mark the consolidation of a new stance. Because affect can either catalyze or derail learning, the classroom ecology—the relational, affective, and institutional conditions of the course— is critical. Cohesive norms (listening, accountability to evidence, space for dissent), transparent criteria for participation, and instructor modelling of vulnerability can keep productive tensions from hardening into resistance. In CP terms, this is the ethical ground of dialogue; in transformative learning terms, it is the facilitative environment for perspective transformation (Freirean praxis on one side, Mezirowian discourse on the other).

However, student-teachers often work within exam-oriented, hierarchical systems; they may worry that critical tasks will invite conflict or be judged as “off-syllabus.” Here the role of the teacher educator is pivotal: calibrating the degree of dissonance, offering low-stakes rehearsal (micro-teaching), and making alignment to curricular goals explicit (e.g., linking dialogic debate to speaking fluency, argument structure, hedging). Such moves reframe CP not as an add-on but as a way to meet existing outcomes more equitably. When programs also provide supportive supervision and institutional signals (e.g., acceptance of alternative assessment artefacts), student-teachers are better positioned to take principled risks and sustain them in practicum and early teaching (Kincheloe, 2008).

Taken together, Freire supplies the ethical–political horizon—praxis, dialogue, social transformation—while Mezirow clarifies the cognitive–affective journey adults undertake as they reassess beliefs. The two frameworks, in concert, explain not only what a CP course teaches but also how it is received, resisted, and potentially internalized. In the CP context, “doing something” with knowledge is central: learning extends beyond the classroom into the broader community, where students may contest entrenched structures; for this to be meaningful, theory must be repeatedly tied to lived experience so that academic work and social action inform one another like two sides of a coin (Jeyaraj and Harland, 2014; Kincheloe, 2008).

### Enacting CP in teacher education: classroom ecology, resistance, and design implications

To integrate CP into ELT curricula means to adjust more than the theoretical stance of educator; it also involves questioning classroom interaction, power relations, and the educator’s identity. For instance, Crookes (2013) argues that CP is essential for preparing English teachers to navigate complex global and local inequalities and to act as agents of change in their classrooms. However, integrating CP into ELT curricula requires more than a theoretical commitment; it necessitates a radical rethinking of classroom interaction, power dynamics, and the role of the teacher. Teachers must be willing to cede control, create spaces for student voice, and model vulnerability—all of which may be unfamiliar or uncomfortable for learners and instructors alike (Akbari, 2008). Furthermore, because many ELT students have been socialized into hierarchical and exam-focused educational systems, they may initially resist the open-endedness and ambiguity that CP entails (Kızıldağ, 2023).

Indeed, pedagogical resistance is a central theme in the literature on CP in ELT. As students encounter unfamiliar pedagogical moves—such as being asked to challenge texts, share personal experiences, or critique institutional norms—they may feel uncertain or even threatened (Norton and Toohey, 2004). Some may question the legitimacy of these methods, while others may fear peer judgment or instructor disapproval. These tensions underscore the importance of what calls the “classroom ecology”—the relational, affective, and institutional conditions that shape how CP is enacted and experienced. When educators fail to attend to these dynamics, CP can inadvertently reproduce the very exclusions it seeks to dismantle. For instance, critical ELT teachers use complex social problems as a vehicle for learning the language in contrast to the traditional ELT teachers who typically select content for its neutrality. The present study focuses on such practitioners, who represent the transformative potential of CP within a traditionally apolitical field like ELT. Akbari (2008) states that there has been an interest in the practical implications of CP recently but it has been only limited to theoretical exploration (Akbari, 2008). In addition, theory has been focused on only student engagement with critical issues, but not about the teachers who actually facilitate learning.

Despite the marginalization of CP in mainstream ELT and the institutional constraints noted above, several studies suggest that CP can foster profound transformation in student-teachers when implemented thoughtfully and responsively. In a study of Turkish EFL teachers, Ulusoy and Dinçer (2020) found that exposure to CP principles helped participants reconsider their assumptions about teaching and begin to envision more democratic and socially responsive practices. Similarly, Canagarajah (2005) emphasizes that teacher education programs that incorporate reflective journaling, dialogic learning, and critical discussion can enable students to critically examine their positionalities and develop greater pedagogical agency.

## Methodology

### Research design and context

This study was conducted within a Master of Arts (MA) in English Language Teaching (ELT) program at a foundation university in Türkiye, a context where English language education—particularly at the tertiary level—is shaped by exam-oriented priorities, standardized curricula, and growing internationalization pressures. In many Turkish universities, English language instruction is still heavily influenced by traditional methodologies that emphasize grammar-based teaching, accuracy, and preparation for proficiency exams such as YDS or TOEFL (Kırkgöz, 2008). Although there has been increasing advocacy for communicative and student-centered approaches, especially in private and foundation universities, the incorporation of CP and social justice-oriented content remains limited (Atay, 2008). Institutional cultures often privilege compliance with centralized standards over pedagogical experimentation, and this can create tensions for instructors seeking to implement inclusive, reflective, or transformative practices.

Against this backdrop, the present research was carried out during a graduate-level course titled Critical Pedagogy in ELT, which by nature introduced challenging content and encouraged examination of classroom power dynamics. This course context was purposefully chosen because its content often confronted students with new, uncomfortable ideas and classroom hierarchies, creating opportunities for significant reflection and perspective shifts (Shor, 1992). The relatively small, seminar-style class setting allowed for in-depth qualitative exploration of each student’s experience. By focusing on this single class over a semester, the study constitutes a small-scale qualitative case study of transformative learning in practice (Stake, 1995). This course met weekly, and students were regularly prompted to reflect on their learning experiences. The design of the study, incorporating repeated reflective journaling and follow-up interviews, enabled the researchers to identify critical moments in the learning process—moments of pronounced confusion, realization, or emotional discomfort. In transformative learning theory, such pivotal moments are known as “disorienting dilemmas,” referring to experiences that disrupt learners’ assumptions and can spark a change in perspective (Mezirow, 1991). By capturing data at multiple points in time, the research traced how students made meaning of these dilemmas and navigated cognitive, emotional, and relational shifts throughout the course. This longitudinal qualitative approach (over the duration of the course) provided rich insight into not just whether students changed, but how that change unfolded in response to the course content and interactions (Merriam and Tisdell, 2016).

### Participants

The participants were eight ELT master’s degree students out of 15 enrolled in CP course. A purposive sampling was utilized to recruit participants as the study involved investigating the participants’ reflective and transformative experiences in working with coursework focused on CP. All students were informed about the research project in the first 2 weeks of the semester and all participation was voluntary. Students who were willing to participate in the research were invited to sign informed consent forms. No exclusion criteria was applied except for the course enrollment. These eight participants who volunteered submitted ongoing weekly reflections and agreed to participate in follow-up interviews. This self-selection process aligns with ethical research in qualitative research, especially in research that could involve sensitive issues like pedagogical beliefs, discomfort, or potential ideological resistance (Creswell and Poth, 2018; Merriam and Tisdell, 2016).

The group of participants included five females and three males with varying personal and professional backgrounds. Of the five females, three were working as instructors at a university English preparatory program. Of the three female instructors, one had a degree in American Language and Literature, aged 45, and the other two participants had degrees in ELT and were 24 and 50 years old, respectively. Of the other two female participants, one was working in a private school and was 23 years, and one was a graduate from a Translation and Interpretation department and was working at a private language course and aged 24.

The three male participants also brought varied experiences. One of them was a 47-year-old graduate of English Language and Literature working at a university. The second one was a 35-year-old graduate of ELT working at a private school. The last male participant was also a graduate of ELT aged 42, who was working as an English teacher at a public school. The variation among the participants in terms of age, educational background, and teaching context substantially enriched the dataset and allowed for examination of different personal and professional identities of participants with respect to their engagement with CP (Table 1).

**Table 1:** Participants’ demographic information.

Participant	Gender	Age	Educational background	Current employment
P1	Female	45	American Culture and Literature	University instructor
P2	Female	24	ELT	University instructor
P3	Female	50	ELT	University instructor
P4	Female	23	ELT	Private school teacher
P5	Female	24	Translation and Interpretation	Private language course instructor
P6	Male	47	English Language and Literature	University instructor
P7	Male	35	ELT	Private school teacher
P8	Male	42	ELT	Public school teacher

### Course context and syllabus

#### Course setting and aims

The research is based on a graduate-level course “Critical Pedagogy in ELT” taught in an ELT teacher-education course. Over a total of 14 weekly sessions, the course had the goals of: (a) building a critical language awareness of how English uses power, identity and ideology; (b) linking the principles of critical pedagogy to the design of pedagogical tasks and assessments in ELT; and (c) engaging students in reflective, dialogic classroom habits of practice that are feasible in high-stakes testing environments (see Table A1 for the week-by-week plan).

#### Teaching approach: dialogic, scaffolded, and problem-posing

The teaching adopted a problem-posing pedagogy with a dialogic stance. The instructor deliberately modeled vulnerability (openly admitting a lack of knowledge, recounting her thought process in making decisions), co-constructed criteria with students, and was explicit about the importance of psychological safety (listening, taking turns, disagreeing). Course materials contained content from core readings (CP, CLA/CDA), authentic artefacts (coursebook pages and extracts, policy texts, media clips), and participant produced materials. There were clearly identified language objectives at every stage so that the critical work was explicitly linked to ELT outcomes.

#### Rationale and implementation of dialogic stance, CLA and CDA

A dialogic, problem-posing stance was adopted because critical language pedagogy at university level benefits from dialogic participation and negotiated norms, which support deeper engagement and perspective-taking. To make explicit how linguistic resources encode representation, Critical Language Awareness (CLA) was incorporated in text-based work aligned with ELT outcomes. Moreover, Critical Discourse Analysis (CDA) was used to structure tasks that examine media, policy, and coursebook discourse so that ideology and power are surfaced while learners practice stance, hedging, and evaluative lexis.

**Figure 1:**
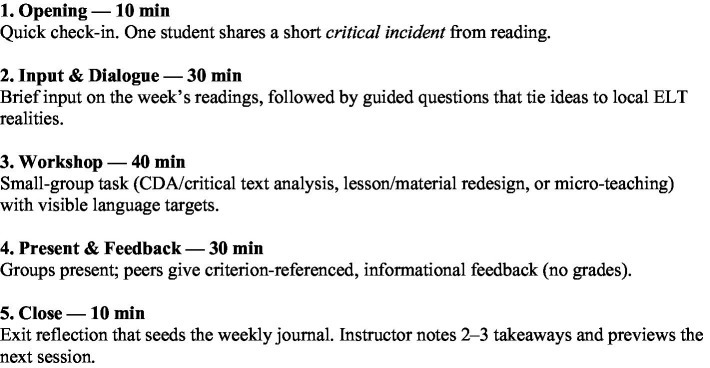
Session flow.

Operationally, the course followed a stable sequence: brief input and guided dialogue, followed by small-group CLA/CDA workshops, presentations with criterion-referenced peer feedback, and exit reflections (Figure 1). Within this sequence, these practices were implemented (1) news critical-literacy workshops using SFL-informed projection analysis; (2) coursebook focusing on stance, voice, and representation, followed by redesign of tasks with explicit language objectives; (3) micro-teaching of the redesigned tasks to rehearse pedagogy; and (4) reflective journals and debrief interviews to trace cognitive/affective shifts in participation and perspective-taking. This sequencing is consistent with published CLA/CDA classroom models and recent “CDA-as-pedagogy” accounts (Huang and Guo, 2024; Chen et al., 2024; Askari and Behdarvandirad, 2025). In practical terms, CLA/CDA anchored Weeks 3–4 and 7–9 (text-analysis and redesign cycles) and recurred in Weeks 10–12 via micro-teaching and peer feedback, while dialogic routines ran every week to stabilize participation and safety norms (see course flow/weekly plan).

### Data collection

Data were collected through two primary qualitative methods: *weekly reflective journals and semi-structured interviews*. Using multiple data sources allowed for triangulation of findings and a more comprehensive understanding of each participant’s transformational learning journey (Denzin, 1978; Flick, 2018). Throughout 14 sessions, data were obtained over weekly intervals through immediate post-session reflective journals (Weeks 1–14) to document in-the-moment cognitive/emotional reactions to course content and activities. After the final course session, semi-structured interviews were conducted to assist with retrospective sense-making across the semester and to further probe the emergent themes from the journals. This sequence facilitated the integration of two forms of reflections to (a) minimize hindsight bias in gathering contemporaneous reflections and (b) triangulate short-cycle reflections with later integrative portraits of change over time.

### Reflective journals (weekly reflections)

During the semester, participants kept weekly reflective journals of each class session, where the reflective prompts were organized using Gibbs’ Reflective Cycle (Gibbs, 1988) for structure and deep reflection. Gibbs’ cycle is a widely recognized and used six-stage model of reflection (Description – Feelings - Evaluation – Analysis – Conclusion – Action Plan) that guides individuals to reflect systematically on their experiences.

Each week, students were asked to reflect on their class experiences, express their feelings during the lesson, evaluate what went well, or challenging, analyze why that happened or felt that way, figure out what they learned or could have done differently, and formulate an action plan for future situations. Sample prompts included: “What challenged your assumptions this week?,” “How do you see power relations operating in your classroom?,” and “In what ways has your thinking about teaching changed since the beginning of the course?” These journals served not only as data but also as pedagogical tools to scaffold reflective thinking.

This structured approach ensured that participants went beyond superficial comments to critically engage with the course material and their reactions to it (Moon, 2004). In practice, this meant the students’ journals captured not only what happened each week, but also why it mattered to them and how they might handle similar issues going forward.

The weekly reflections served multiple purposes in the study. First, they provided immediate, first-person accounts of the students’ cognitive and emotional responses to the CP content each week. Because the content often challenged their preconceived notions (for instance, about teacher/student roles or sociopolitical issues in ELT), students frequently grappled with discomfort or surprise in their writings (Akbari, 2008). These written accounts allowed identification of any emerging disorienting dilemmas as they occurred. Second, the longitudinal nature of the journals (accumulating over several weeks) made it possible to observe changes or development in thinking over time.

Participants wrote their reflections in the language of their choice (English or Turkish). Although the course was conducted in English, it was important that the reflection process itself be in a “safe” language for the participant (Canagarajah, 2005). Therefore, students were explicitly allowed to write in their native language (Turkish) if it enabled them to express their thoughts and feelings more freely. This choice aimed to create a safe environment where language barriers would not hinder honest reflection (Cummins, 2001).

### Semi-structured interviews

At the conclusion of the course (after several weeks of reflections had been collected), each participant took part in an individual face-to-face semi-structured interview. The interviews were scheduled during the final week of classes or shortly thereafter, each lasting approximately 20–30 min. A semi-structured format was chosen so that key topics would be covered with every participant, while still allowing flexibility to follow the participant’s lead and probe interesting points in depth (Kvale and Brinkmann, 2009). An interview protocol was prepared, consisting of open-ended questions and prompts that built upon the content of the reflective journals. The interview protocol included questions such as: “Can you describe a moment during the course that shifted your perspective on teaching?,” “What emotions did you experience during those shifts?,” and “Have you attempted to apply any of these new perspectives in your teaching?” Interviews lasted between 20 and 30 min and were audio-recorded with participants’ consent. Both data sources allowed for triangulation and facilitated a rich understanding of participants’ transformative learning processes.

Consistent with the effort to maintain a comfortable environment, the language of the interviews was chosen by the participants. Each interviewee was given the option to converse in English or Turkish. Allowing participants to use their preferred language helped reduce power imbalances and anxiety (Barkhuizen, 2011).

The interviews were conducted by researchers in a private setting on campus, usually just before or after a class session to maximize convenience. All interviews were audio-recorded with permission. The recordings were subsequently transcribed verbatim for analysis. During transcription, any identifying information (names of people, specific program details) was replaced with pseudonyms or generalized descriptions to protect confidentiality.

### Data analysis

To guide the students’ reflective writing, Gibbs’ Reflective Cycle (Gibbs, 1988) was introduced and employed as a pedagogical tool. This six-stage model (description, feelings, evaluation, analysis, conclusion, action plan) provided a structured format for weekly journals and helped ensure that students engaged in critical, in-depth reflection on their experiences. The reflective structure itself was not used as an analytical framework but rather as a scaffold to enhance the quality of student reflections.

Once the data (weekly reflections and interviews) were collected, all qualitative content was analyzed using Braun and Clarke’s (2006) six-phase thematic analysis framework. This inductive approach allowed for the identification of patterns across data sources, focusing on cognitive, emotional, and relational shifts. Thus, while Gibbs’ model structured the data production process, Braun and Clarke’s method provided the analytical lens for identifying themes and meaning-making trajectories within the data.

Thematic analysis was chosen since it is a systematic but flexible approach to identifying patterns of meaning across a dataset. The non-deductive approach and lack of relation to a specific theoretical stance makes this method a good fit for exploring the students’ range of personal experiences (Nowell et al., 2017).

An inductive approach was taken for this study, that is the themes were heavily rooted in the actual data of the participants taking part, as opposed to theory and hypotheses from the previous literature (Braun and Clarke, 2006). The analysis grew from Braun and Clarke’s six steps: familiarization, initial coding, developing themes, reviewing themes, naming themes, and writing up. NVivo software supported data management and organization of codes.

Intercoder reliability strategies were also utilized to ensure credibility. Two researchers independently coded the data, then discussed any identified discrepancies that occurred with each segment of data until consensus was reached (Barbour, 2001). Reflexivity was maintained throughout the process, acknowledging the researchers’ positionalities and potential influence on data interpretation (Finlay, 2002).

Moreover, both concurrent weekly journals and end-of-course interviews were implemented and triangulated across these data sources throughout analysis to reduce demand characteristics. Negative-case analysis to check for disconfirming evidence and retained verbatim excerpts to present the participants’ voice in analysis were conducted. Researcher reflexive memos documented assumptions and analytic decisions across phases (Braun and Clarke, 2006). Together, these procedures increase trustworthiness of the findings that observed changes manifest as shifts in sense-making rather than a result of participants merely motivating their person to align with researcher-inferred expectations.

### Ethical considerations

Ethical approval was received from the university’s institutional review board. Participants provided informed consent and were assured of confidentiality and voluntary participation (British Educational Research Association, 2018). They were provided with pseudonyms and their data were stored safely (Creswell and Poth, 2018).

## Results

**Figure 2:**
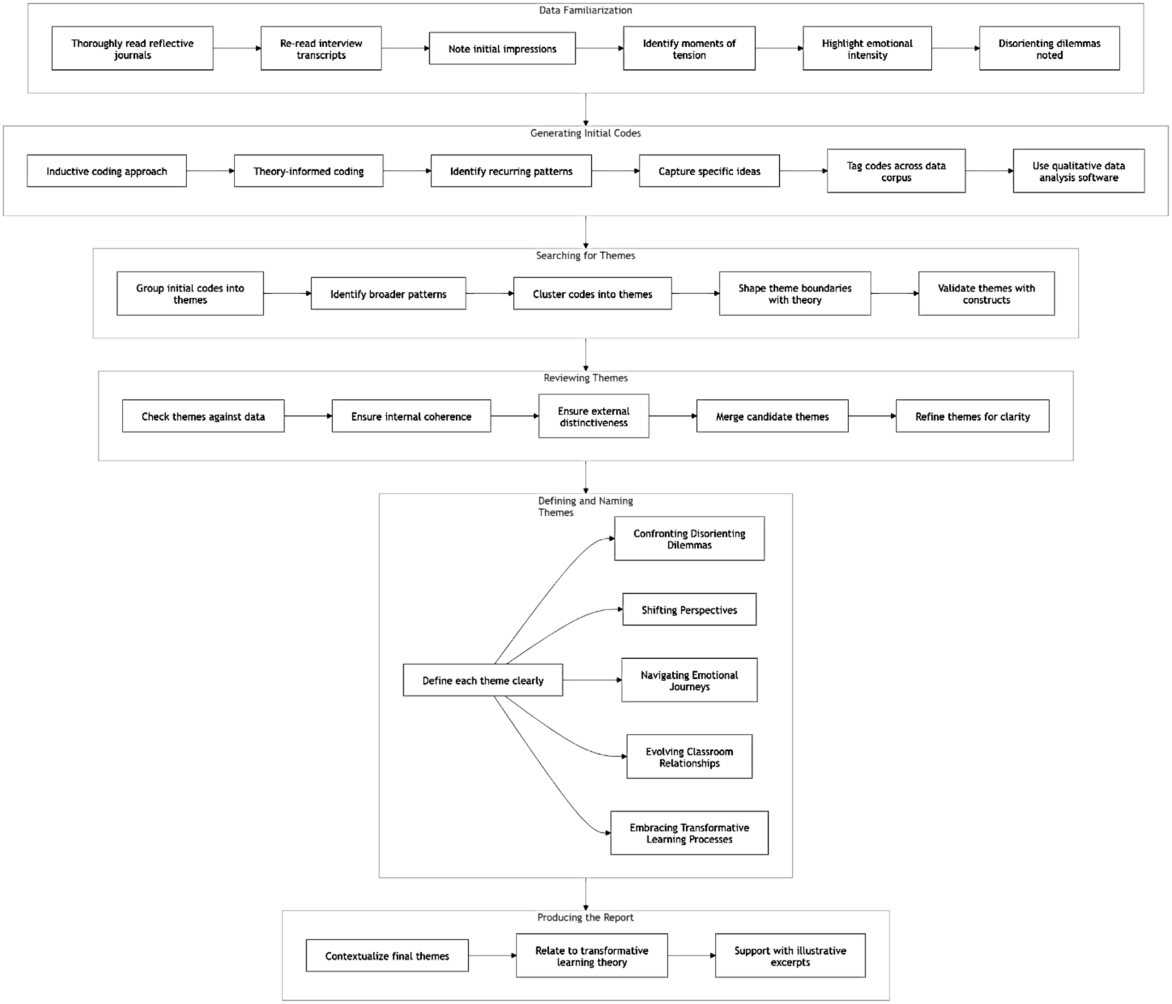
Thematic analysis process (Braun and Clarke, 2006).

Following Braun and Clarke’s six-phase approach to thematic analysis, five overarching themes were identified that encapsulate the participants’ experiences: *Confronting Disorienting Dilemmas, Shifting Perspectives (Cognitive Transformations), Navigating Emotional Journeys, Evolving Classroom Relationships, and Embracing Transformative Learning Processes* (see Figure 2). These themes emerged through a blend of inductive analysis and theoretical grounding in transformative learning. They reflect the complex, dynamic, and multi-dimensional nature of the participants’ developmental processes in response to CP. *In Phase 1 (Data Familiarization),* the researchers began by thoroughly reading and re-reading the reflective journals and interview transcripts to become immersed in the data. During this process, initial impressions were noted, with particular attention to moments of tension, insight, or emotional intensity. For example, several participants wrote about moments that challenged their beliefs about teaching English as a neutral act—these instances were noted as potential disorienting dilemmas. *In Phase 2 (Generating Initial Codes),* using both inductive (data-driven) and theory-informed (Mezirow’s transformative learning theory and Freirean CP) coding approaches, the researcher identified recurring patterns and meanings. Each code captured a specific idea, such as: *“Shock at CP readings,” “Questioning textbooks,” “Guilt about past practices,” “Reimagining teacher role,” “Writing as self-reflection.”* These codes were tagged across the data corpus using qualitative data analysis software or manual coding in tables. *In Phase 3 (Searching for Themes),* the initial codes were then grouped into potential themes that reflected broader patterns in the data. For instance: codes like “*shock,” “discomfort,” “questioning neutrality”* clustered into the theme *Confronting Disorienting Dilemmas*. Codes such as *“new teacher identity,” “lesson planning with critical aims,” “questioning power in the classroom”* were grouped under *Shifting Perspectives.* At this stage, theoretical constructs (e.g., Mezirow’s phases of perspective transformation and Freire’s notion of praxis) helped shape and validate theme boundaries. *In Phase 4 (Reviewing Themes),* the themes were checked against the data again to ensure they were both internally coherent (the data within each theme fitted together meaningfully), externally distinct (themes captured different aspects of experience). Some candidate themes were merged (e.g., “emotional resistance” and “emotional growth” became Navigating Emotional Journeys), while others were refined for clarity. In *Phase 5* (Defining and Naming Themes), each theme was defined to clearly capture the essence of what it represented in the participants’ learning journeys: “*Confronting Disorienting Dilemmas*” captures the initial shock or disruption, *“Shifting Perspectives*” reflects the cognitive restructuring process, *“Navigating Emotional Journeys”* highlights the affective component of learning, “*Evolving Classroom Relationships*” shows how participants began to redefine power and engagement in their imagined or real teaching and *“Embracing Transformative Learning Processes”* captures their movement toward action and praxis, consistent with Mezirow’s final phases of transformation. *In Phase 6 (Producing the Report),* the final themes were contextualized in relation to transformative learning theory and CP literature, supported by rich, illustrative excerpts from participants’ reflections and interviews.

In the excerpts below (from weekly reflective journals and post-course interviews), participants are referred to by participant codes (P1, P2, etc.) to ensure anonymity. Each theme is illustrated with rich, participant-generated examples accompanied by analytical commentary on shared patterns, tensions, and contradictions across the group.

### Confronting disorienting dilemmas: catalysts for critical reflection

Participants began the course by encountering challenging ideas and experiences that unsettled their prior beliefs about teaching and learning. These “disorienting dilemmas” (Mezirow, 1997) functioned as strong promoters of reflection. In Mezirow’s transformative learning theory, disorienting dilemmas occur when a person encounters an experience that does not align with what they know, leading to critical self-reflection. In the example, P1 describes her reaction to Freire (1970) implication that all education is inherently political, prompted her to deeply rethink her previously unquestioned assumptions about the neutrality of English language instruction.

“I always thought of English teaching as a neutral, skills-based exercise. Freire paints a perspective of education that is inherently value-laden, and through this reading, I was able to question my initial assumption. It was then that I began noticing how some of the texts I actually use represent particular cultural narratives that (implicitly) exclude other narratives. I started to think more about the meaning that classroom content can have in framing students' feelings of belonging and engagement. This was hard for me to process, the fact that my teaching could be limited in such an insidious way, but the reflection allowed me to be more aware of the bigger picture of my instructional decisions.” (Reflective Journal, Week 2, P1)

This excerpt demonstrates an initial phase of transformative learning, in which the learner begins to critically reflect on previously unexamined assumptions (Mezirow, 1997). P1’s reflection shows a small, but important, upward shift in critical consciousness: from thinking about English language teaching as not political to thinking about the cultural and social aspects of what is included in the content. Rather than placing a binary on right and wrong, the participant recognized that educational decisions can be complex and tend to include or exclude potential perspectives. This result may be considered as a moment of critical awareness; Mezirow (2000) notes the process of “becoming critically aware of one’s assumptions and expectations and those of others,” is the first step to deeper engagement with pedagogy. It also illuminates the disturbing emotions and the growing sense of responsibility she felt for what she had previously taken for granted. Perhaps equally illustrative is the example reported by P2 in her interview where she also had to reconsider an aspect of her teaching practice because of a class discussion about the representation of gender roles in commonly used EFL textbooks:

“During that discussion, I suddenly became aware of how often the materials I use portray very traditional gender roles—fathers going to work, mothers staying at home. I had never really questioned it before. I guess I assumed that was just how things were, or maybe I didn’t want to complicate my lessons. But hearing others talk about it made me realize that these depictions can shape how students see the world and their place in it. Since then, I’ve started looking at my materials more critically—not just in terms of grammar and vocabulary, but in terms of the messages they carry.” (Interview, P2)

This excerpt exemplifies the initial phase of transformative learning, where a disorienting experience prompts critical reflection on previously unexamined assumptions (Mezirow, 1997). In this case, P2 begins to recognize the implicit social and cultural messages embedded in classroom materials—an awareness that was previously absent. Her reflection illustrates a shift from a purely content-oriented focus toward a more critically engaged pedagogical stance. While the transformation is still in its early stages, the narrative demonstrates a growing capacity to interrogate normative discourses within educational content, which is a hallmark of the CP framework (Freire, 1970; Brookfield, 2017). Moreover, the excerpt highlights how dialogic encounters with peers and course content can serve as catalysts for unsettling and reconfiguring long-held professional assumptions. This kind of critical introspection, especially when accompanied by supportive classroom discourse, lays the foundation for sustained cognitive and pedagogical change.

Such moments of sudden dissonance were common across the weekly reflections. Several participants noted feeling “confused,” or “overwhelmed,” when first grappling with CP concepts that challenged traditional ELT practices. This sense of confusion and imbalance is characteristic of disorienting dilemmas because the experience conflicts with one’s former ways of knowing.” The data suggest that encountering these dilemmas was a necessary first step in the transformative journey. P3, for instance, wrote about her initial struggle to reconcile her long-held views with the unfamiliar terrain of CP:

“At first, I wasn’t sure what to make of the ideas we were discussing. I’ve always thought of my job as teaching correct grammar and helping students pass their exams. But then we started talking about how language teaching can either maintain or challenge inequalities. That really confused me. I began to wonder if I had been ignoring the broader impact of what I do in the classroom. It felt like everything I believed about being a teacher was being questioned all at once.” (Reflective Journal, Week 3, P3)

This excerpt demonstrates how P3’s confrontation with CP concepts triggered cognitive dissonance, which in Mezirow’s (1997) terms marks the onset of transformative learning. Her confusion and uncertainty reflect the destabilization of her prior teaching identity—a discomfort that, while unsettling, catalyzed deeper self-inquiry. Such affectively charged encounters with unfamiliar paradigms can push learners toward critical reflection (Taylor, 2008). Notably, P3 does not yet articulate an alternative vision of practice, but the act of questioning her prior assumptions signals a crucial entry point into the reflective process. As Brookfield (2017) argues, these uncomfortable moments are not barriers but opportunities through which educators begin to re-evaluate their roles, values, and classroom choices.

For P3 and others, such eye-opening events triggered critical questioning of previously unexamined beliefs. However, not all participants experienced this phase with the same intensity. A few, such as P4, who had prior exposure to sociolinguistics, reported being “less surprised but still challenged” by the content, indicating that the impact of a given dilemma varied depending on one’s background knowledge and dispositions. This aligns with Mezirow’s statement that not every challenging event will be transformative for every learner. However, for the group as a collective, facing disorienting dilemmas in the course, created the instability to initiate reflection and change that facilitated deeper learning. As Mezirow (1997) theorizes, the early shock “stimulates self-reflection because beliefs and values have been challenged,” which can be seen again in the participants’ statements.

### Shifting perspectives: cognitive transformations in understanding teaching

After these early dilemmas, participants revealed considerable cognitive transformations in understanding language teaching and their position as teachers. By the end of the course, through ongoing reflection and whole-class conversations, they began to challenge their old beliefs and to develop more critical, broad-ranging perspectives on English language teaching. In other words, these reflections indicated a shift from procedural thinking, focused on how and what to do in the lesson, to critical consciousness about the pedagogical choices they were making as they began to engage with the sociopolitical dimensions of language teaching. For example, P5 mentioned that shift in her journal:

“I used to plan lessons only focusing on grammar points, but now I find myself asking why we are learning this. I realize English teaching can either reinforce the status quo or challenge it. I’d never asked those kinds of questions before, and it really changed my perspective on teaching.” (Reflective Journal, Week 6, P5)

This reflection marks a clear transformation in how P5 perceives the nature of teaching. Rather than treating ELT as a neutral, skill-based enterprise, she begins to interrogate its ideological underpinnings—echoing Freire’s (1970) notion that education is inherently political. The statement also illustrates what Mezirow (1997) terms a “perspective transformation,” in which learners reassess their frames of reference and move toward more inclusive, critically informed ways of understanding their professional roles.

Across the dataset, participants described similar moments of intellectual repositioning. The process was not merely additive (learning new information), but transformative—prompting participants to reframe prior understandings and reconceptualize their teaching goals. Several participants wrote about recognizing how curriculum materials, classroom discourse, and institutional policies could implicitly sustain inequalities. These realizations often emerged not through isolated readings but through sustained reflection, peer exchange, and instructor-guided discussions—aligning with transformative learning theory’s emphasis on dialogic meaning-making (Cranton, 2011; Taylor, 2008).

Another example of a cognitive transformation was from P6, who expressed a radical change in his understanding of what it is to be an English language teacher. In his Week 6 journal, he reflected as follows:

“Before this course, being a good teacher meant being able to follow the syllabus as closely as possible and making sure my students passed their exams. But as I gradually engaged more deeply in the readings and in our class discussions, I started to question the kind of education I was providing. I began to see teaching English could imply much more than vocabulary and grammar. I find myself being more thoughtful about the content I use and how they could influence students' sense of identity and belonging.” (Reflective Journal, Week 6, P6)

This reflection demonstrates a significant epistemological transformation in the participant’s conceptualization of nature and purpose of language education. P6’s growing awareness of how instructional materials may affirm or marginalize the identities of his students parallel the existing questions in critical applied linguistics about the restatement of dominant ideologies within the curriculum (Pennycook, 2001; Pennycook, 2010; Gray, 2010). By questioning what voices exist – or do not exist – in his teaching, P6 represents a shift away from a technical-rational conception of ELT towards a critical-ethical stance, concerned about representation, equity, and cultural inclusion (Kumaravadivelu, 2006; Akbari, 2008). In addition, his remarks demonstrate an increasing commitment to the praxis dimension of CP, which entails a dialectical reflection-in-action process in the pursuit of social justice (Freire, 1970; Giroux, 2011). With these remarks, P6 further models the types of critical consciousness CP seeks to foster, as he identifies language teaching as socially and ideologically loaded. The developing position he displays illustrates not only a shift in thinking but also a moral and professional re-positioning of the teacher role from a transmitter of knowledge to an agent of change.

Through the interviews, participants were able to articulate how their view of teaching shifted, with many of the participants explicitly using terms like “eye-opening” or “now I understand...” when drawing on their beliefs both prior to and after the course. P7 explained:

“Before, I thought my job was to teach language skills. After this course, I see myself as helping students question social issues through English. I am not just a language teacher, but rather a mentor, to help them become critical thinkers.” (Interview, P7)

This response is remarkable, with regard to a development in P7’s professional identity and pedagogical awareness. In fact, the development is reflective of what Cranton (2011) refers to as a “deep shift in perspective” that makes the development of open, critical and reflective ways of thinking possible. As the participants were afforded opportunities to engage with critical pedagogical concepts, it appeared they began to engage with education less as a neutral enterprise for learning syllabic learning -and more as a source of social change. This is in keeping with Kincheloe (2008) who suggest educators should come to terms with their work in a broader sociopolitical reality in a way, in order to dismantle oppression and inequality.

It is worth noting that while all participants showed cognitive change, the degree of change and reasoning. Teachers that had more direct experience in the field made many references to specific pedagogical strategies changed in the present - a teacher writing new lesson plans about social justice topics, teachers altering their dialogical methods to allow the learners to feel they found their voice. On the other hand, for participants without any teaching experience, many abstracted their purpose for practice all, thinking they would engage in process somewhere down the road. This variability points to the possible influences of some contextual factors, including professional experience and teacher autonomy on the level of engagement with critical awareness. (Mezirow, 1997; Taylor, 2008).

A subtle tension exists in the experience of P8, who expressed areas of intellectual alignment with the theories behind CP, but retained some uncertainty about translating it into practice:

“These ideas are good, but how far can we actually go in practice given the pressures for exams, and limits to the course?” (Interview, P8)

P8’s comments illustrate a contradiction that many participants expressed—the ideological alignment to the aims of CP vs. the limits imposed by the challenging institutional and traditional practices of a standardized system. This contradiction also further highlights what Brookfield (2006) refers to as the “praxis gap,” or the struggle to align critical aspirations with institutional restraints. Nonetheless, a strong theme among participants was without a doubt genuine cognitive growth, represented as a change in their assumptions about the function of language teaching in advancing equity, inclusion, and critical consciousness. In fact, this change follows the idea as embedded in transformative learning theory that sustainable equitable change is established through a persuasive shift in the way people perceive themselves, and their roles (Mezirow, 1997; Cranton, 2016).

### Navigating emotional journeys: affective responses to critical pedagogy

For these students, engaging in CP was not only a theoretical experience, but an emotional experience. As students struggled with questioning their assumptions about teaching and learning, they also experienced varying affective responses—ranging from anxiety and guilt to excitement and a sense of empowerment. Throughout the first weeks of the study, many felt uneasy, defensive, or guilty when confronted with what CP implies. P4 wrote in her Week 3 journal about the emotional struggle of questioning her assumptions about teaching:

“When we discussed how teaching English could sometimes harbor certain cultural values, I started to think about how I may be disregarding the cultural identities of my students. It was not something I ever gave much thought to before, and just recognizing this prompted me to question my thinking further. It felt a little uncomfortable at first but opened a new awareness that I now value.” (Reflective Journal, Week 3, P4)

P4’s response highlights the emotional labor associated with CP. Her initial discomfort, which resulted from conversations in the class about cultural representation, led her to become more aware of remaining approaches to pedagogy that may ignore or marginalize students’ cultural identities. In this way, P4’s shift in reflection demonstrates the emotional aspect of transformative learning, whereby dissonance could serve as a prompt for development (Mezirow, 1997). Rather than abandoning being uncomfortable, P4 had a developing awareness as she actively engaged in teaching more intentionally and inclusively. This reflection resonates with Taylor (2008), when she suggests that emotion is a salient and necessary element of transformative learning. P4’s experience further illuminates the connection between emotion and cognition within critical reflection, suggesting that the emotional dissonance (when contextualized in a safe learning space) can promote a willingness to change and reoriented practice.

Yet, as the course progressed, the participants also described how these uncomfortable emotions took the form of more positive emotions of growth, empathy, and empowerment. Together with the instructor and each other’s support, the initial anxiety was gradually replaced with a feeling of “motivation to change.” P8, in her interview, described her emotional experience as a “roller coaster,” saying:

“In the beginning, I felt very tense---like I was being judged or that my past pedagogical practices were being questioned. As we began to share our reflections and practiced listening to one another, I came to realize we were all on the same page, which helped warm me up. The guilt I felt about previous practices began to transform slowly into a feeling of commitment. I started to think, ‘Okay now that I know better, I want to do better.’ It felt emotionally intense, but it also felt really empowering.” (Interview, P8)

This narrative is an example of a journey from defensiveness to what Mezirow (2000) calls transformative learning through critical reflection in which the learner not only recognizes dissonance but works to reconstruct meaning in response to that dissonance. The journey of P8 illustrates this movement from a state of vulnerability to agency—a change that has been documented in studies of CP in which peer support and emotional validation are important factors in reflective practice (Zembylas, 2013; Taylor and Cranton, 2013) The participant’s comment that “we were all in the same boat” points to the communal aspect of critical reflection: it is rarely an individual change, but rather a change fostered in dialogic contexts (Brookfield, 2017). The safe and inclusive environment established in the course seem to have diminished risks of emotional vulnerability, while enabling the building of what Cranton (2016) calls “authentic relationships” that are important to transformative learning. Over time, and through the ongoing reflection and dialogue, emotional distress can shift to what one participant referred to as “productive passion.” This change is evident when we think of the emotional arc in transformative learning which goes from disquiet to purposeful motivation (Taylor, 2008). For example, in her Week 10 reflection, P1 wrote:

“At first, I was unsure, and a little overwhelmed, with all of the new ideas. But, as I continued to think and write each week, something changed. I realized I was becoming emotionally invested in doing right by my students. I feel a deep responsibility now to provide a fair and supportive learning environment for each of them—not just academically, but emotionally and culturally too.” (Reflective Journal, Week 10, P1)

P1’s account signals a turning point in her engagement with the course material—where emotional labor is rechanneled into a sustained ethical commitment. This shift reflects what Mezirow (1997) conceptualizes as a reintegration of new perspectives into one’s professional identity. Rather than avoiding discomfort, P1 embraces it as a source of insight and moral clarity. Her use of the phrase “emotionally invested” suggests a deep internalization of the course’s critical pedagogical principles, marking a progression from awareness to action. This form of emotional engagement, as Zembylas (2015) argues, is integral to CP because it fosters affective solidarity with learners and motivates transformative praxis.

By the conclusion of the course, most participants associated CP with positive affective states such as inspiration, empowerment, and a heightened sense of moral purpose. The emotional journeys undertaken by participants thus both complicated and enriched their learning, underscoring that transformation is as much a matter of the heart as of the mind (Taylor, 2008). The tension between discomfort and growth remained a salient theme—while not every disturbing feeling was fully resolved for every participant, by course end all acknowledged that these emotional trials were integral to their development as critical educators.

### Evolving classroom relationships: reconfiguring power and identity

In tandem with cognitive and emotional shifts, participants experienced significant relational shifts in how they viewed their roles and relationships in the classroom. As their understanding of CP deepened, they began to reimagine the teacher–student dynamic from a hierarchical one to a more dialogic and egalitarian model. Many came to see teaching as a collaborative endeavor and started valuing student voice and agency more than before. This theme emerged very clearly in the reflection of those participants who were teaching at the same time as they reflected, or those who were drawing on previous teaching experiences. For example, P7 reflected in his Week 8 journal of altering the way he was teaching in his classroom:

“I stopped lecturing so much and started listening. I asked my students to bring up topics that were meaningful to them. The energy in my class changed completely - students opened up when I started to treat them like partners in learning. I could see their confidence grow when they realized their thoughts and opinions mattered in our class.” (Reflective Journal, Week 8, P7)

This practical shift by P7 – from teacher-centered to a place of collaborative dialogue – represents the relational transformation many wanted. After allowing students more choice and voice, he noticed a lot more student engagement and trust growing in his class. These changes are representative of the principles of CP, which supports creating learning spaces where students and teachers are in a more reciprocal relationship, and everyone has an equal say. P6 described in his interview a sense of reconceptualizing his approach to teaching; he said:

“I’ve always thought a 'good teacher' needed to have the kids under strict control and always follow teacher direction. I now realize that learning only happens if I step down from the podium. I believe that building a culture of learning amongst us is the building block of learning. It's a total shift from my previous thinking of needing to be the main authority figure at all times.” (Interview, P6)

P6’s intention to change himself from singular authority to co-learner implied some alteration in the power relationship. In this case, the participants were becoming increasingly critical of the traditional top–down teacher role. They used words such as dialogue, respect and empathy to characterize the people they hoped to be as teachers. Many participants said they began to listen more diligently and exercise empathy towards students. For example, in her interview, P2 reflected on the importance of knowing her students as individuals:

“I used to feel that it is better to have distance between my students and I. I thought personal things were not my business. Now, I think it is important to know who my students are and how their lives and perspectives shape them. I think I have included genuine trust in my philosophical approach to being a teacher. I listen to my students' stories and opinions.” (Interview, P2)

This new ethic of care and respect for others represents a significant change in the relationship by reconceptualizing participants’ perceptions of students from passive knowledge recipients to whole beings and active participants in learning. While engaging with the course materials and working with each other, many participants began to think about their teaching roles in a more dialogic and collaborative way, supporting a CP of co-constructed learning (Freire, 1970; Shor, 1996). However, as noted throughout the course, this type of relational transformation is not without tensions or uncertainty. A number of participants recognized general ambivalence towards how to enact shared authority while continuing to manage a productive classroom. P4 provided an example of this situation particularly evident in her reflections. P4 had considerable excitement and willingness to embrace a more democratic teaching model but was also concerned about the reality of more shared authority. She reflected in her interview:

“I want a democratic classroom, but I'm afraid if I give up too much authority, my students will not respect me and I will lose control. There is a fine line between empowering students and creating chaos. I am still determining how to walk that line.” (Interview, P4)

P4’s comments highlight the complex pedagogical dilemma that can emerge when teachers begin to think about changing the established power dynamics of education. She received and embraced the notion of dialogical practice and shared responsibility, while also expressing concern that too much dialogical practice would negatively impact her professional legitimacy or integrity in the practice of teaching. This dilemma parallels what Hooks (1994) refers to as the challenge to teach to transgress and exist within the boundaries of institutional culture - the personal desire for liberatory practice collides with the logistical reality of confined space in a classroom and the limitations of institutional expectations.

A persistent issue that emerged from participant accounts was the conflict between ideals and implementation. Many participants recognized their substantial desire for mutuality and trust with their students, while also recognizing their responsibility to establish limits, foster accountability, and identify roles. Although P4’s anxiety may have derived from an isolated experience, it captured a larger theme across the cohort: the recognition that transformative learning is something that requires intention, but also the ability to be strong when faced with the complexity of the classroom.

Nevertheless, this ambivalence did not negate the broader trend toward relational empowerment. Participants widely expressed a commitment to fostering more inclusive, empathetic, and student-centered environments—even if the path toward such transformation remained unfinished and evolving. This aligns with Mezirow’s (2000) assertion that transformation is not a linear process but a recursive cycle of reflection, experimentation, and revision.

All in all, participants left the course with a vision of teaching that involves working with students rather than doing to students, indicating a profound shift in professional identity. This relational reorientation complements their cognitive shifts: as they came to question power and oppression in society, they also began to redress power imbalances in their own classrooms, however modestly. In summary, participants were learning to “walk the talk” of CP in their relationships, striving to embody the role of the teacher not as authoritarian figure, but as facilitator and fellow learner.

### Embracing transformative learning processes: from reflection to praxis

By the end of the semester, participants had not only changed in outlook and relationships, but also actively engaged in processes of reflection and action that signify transformative learning in motion. A hallmark of transformative learning is that learners critically reflect on their assumptions and begin to make changes based on new understandings. In this study, the weekly reflective journals and the ongoing class dialogues were vital in facilitating such critical reflection. Participants frequently commented on how the structured reflection process was instrumental in consolidating their learning. P1, for example, noted in her final journal:

“Writing each week forced me to be honest with myself. Looking back at my first entry, I can trace how my thinking evolved. I’ve become someone who constantly questions why I do what I do in the classroom. Seeing my own transformation over the weeks on paper has been astonishing.” (Reflective Journal, Week 14, P1)

This reflective practice helped P1 and others progress through the stages of transformative learning – from recognizing a disorienting dilemma, through examining one’s own assumptions, to formulating new approaches. Many participants remarked that the act of journaling and sharing in discussions made them more intentional and critical in their daily thought processes. Participants in this project were not just absorbing theories but actively interrogating how this theory applied to their own contexts. It is important to note that the participants did not stop reflecting, but rather began to translate their newly gained perspectives into actions, signaling the beginning of transformative practice. In interviews, several identified specific actions or intentions that had emerged from the course. P5 recalled that

“I redesigned a lesson plan that included a debate on a social issue because the course made me feel empowered to try something that matters, as opposed to just following the syllabus. I realized that I could begin to incorporate social issues in my English lessons to make those lessons more relevant for students.” (Interview, P5)

Moreover, P6 volunteered in his interview:

“I even went to the administration at my school to start an English club where students can talk about things like racism and gender equality in a safe space. It’s my way of enacting CP at my school.” (Interview, P6)

The examples also illustrate how participants were beginning to move towards what Mezirow (1997) would refer to as trying on new roles and developing plans of action related to their transformed perspectives. Perhaps most importantly, there was a shift in participants’ sense of agency, as they came to see themselves as active change agents as opposed to curriculum deliverers. P3, in her interview, indicated the transformation she experienced:

“This course changed how I think and how I will teach. I'm not leaving with just new ideas, but with a whole new way of thinking. I feel a responsibility now to continue to question the current situation and empower my students to do the same in every class I teach.” (Interview, P3)

Her comment exemplifies how the participants internalized a continued commitment to critical reflection and social justice – they consider transformation to be an evolution rather than a single event. While enthusiasm was at a peak, there was an acknowledgment from some participants that they would face challenges in enacting their transformative learning. For example, P8 expressed enthusiasm about the possibility of introducing critical issues into his classes, while also identifying feeling.

“I am excited about talking about critical subjects in my classes, but I hesitate because I worry about what colleagues or parents in more conservative schools may think or say. I worry they might not support this kind of teaching approach.” (Interview, P8)

Such remarks acknowledge that translating CP into practice can be constrained by institutional contexts and is, itself, a learning process. Nevertheless, the prevailing sentiment was one of determination to overcome these barriers. Participants’ experiences align with the notion that CP necessitates balancing reflection with action as educators learn to cope with the unexpected. Indeed, CP explicitly enables students to act upon and use their knowledge for self and social transformation, and our participants demonstrated this by taking initiative in their spheres of influence. They anticipated that the impact of their transformative learning would extend beyond the graduate classroom – an expectation consistent with the idea that CP’s transformation “is unlikely to end in the classroom but will impact the wider community.”

For these ELT graduate students, the course CP in ELT became a springboard for ongoing transformative practice. Through continual critical reflection, dialogue, and experimentation, they began the work of reintegration – incorporating their new perspectives into their professional and personal lives. In sum, the findings illustrate a group of teachers who have not only transformed their thinking and feeling but are now ready to transform their practice in order to pursue greater equity and social justice in language education. Each participant’s experience was different; however, collectively their stories support the idea that engaging with CP can be fundamentally transformative – intellectually, affectively and in relation to the relationships that underpin the experience of education.

## Discussion

This research examined the transformative learning journeys of graduate students in an ELT course on CP. The findings illustrate a multilayered change process that occurred cognitively, emotionally, and relationally, resulting in conscious action toward socially responsive teaching. The findings, situated within Mezirow’s transformative learning theory (Mezirow, 1997, 2000) and Freirean CP principles (Freire, 1970; Giroux, 2011), demonstrate that embedded opportunities for reflection and dialogical engagement can meaningfully shape teacher identity and practice.

One of the most prominent findings is the role of disorienting dilemmas as starting points to transformation. There were particular moments that disturbed their previous understandings of the neutrality of their ELT– like questioning the representations in their textbooks or highlighting implicit cultural biases. These findings echo Mezirow (1997) claim that critical reflection often arises from an experience that challenges existing assumptions. Similarly to Taylor (2008) comments, these moments were more than cognitive interruptions, they involved emotional disturbance; often manifesting as guilt, confusion or vulnerability. This confirms Zembylas (2013) argument that emotions are not peripheral to transformative learning but rather essential to the process.

As participants progressed through cognitive reappraisal and emotional processing, they began to reshape their identities as transmitters of knowledge to facilitators of inquiry and empowerment. This change was consistent with Cranton’s (2016) description of moving toward authenticity and enacting practices that aligned with their revised beliefs and values. This shift occurred as they engaged in sustained reflective practices—through journaling, peer conversations, and experiential learning exercises. These structured reflective practices are consistent with Farrell (2015) and Brookfield (2017) recommendations for developing critical awareness and pedagogical identity in language teachers.

Moreover, the shifting classroom relationships of participants illustrate a major shift in power relations. Many wanted to think of the teacher-student relationship as a dialogical and reciprocal relationship rather than hierarchical—a vital aspect of Freirean pedagogy (Freire, 1970; Shor, 1996). The relational shifts mentioned in the findings such as creating opportunities for student voice and developing trust suggest a shift towards democratic classrooms that are responsive to learners’ identity and agency. For example, P4 expressed fear of losing authority, indicating the potential for ambiguity in shared responsibility; by no means is the relationship purely reciprocal. This fear echoes the “praxis gap” (Brookfield, 2006) in which educators acknowledge and give intellectual endorsement to CP but have difficulty operationalizing it in institutional contexts governed by standardized curricula and norms of classroom management.

As participants engaged with CP, they also committed themselves emotionally to the success and well-being of their students—an idea echoed by Benesch (2001/2017) who asked educators to attend to the affective aspect of teaching. With their emotions shifting from guilt and anxiety to empowerment and accountability, participants embodied an expanded ethic of care that integrated emotional, cultural, and social responsive ELT practices. This shift is consistent with research in second language teacher education that underscores teachers’ emotions as factors in the formation of identity and agency (Gao et al., 2024; Lemarchand-Chauvin and Tardieu, 2018).

Notably, the research also adds to the growing number of studies reconceptualizing teacher learning as relational and contextual. The fact the participants’ willingness to act – through redesigning lessons or suggesting a project beyond the curriculum offered a clear example of movement from critical reflection to a more active or transformative praxis (Mezirow, 2000). This aligns with Kumaravadivelu (2012) conception of the teacher as a critical reflective practitioner capable of negotiating and resisting the structures of language education. This act of moving toward action further supports Kincheloe (2008) position that a CP ought to position educators not just as interpreters of the world but changers of it.

Still, the participants’ narratives demonstrate the inequality of transformative learning. For example, some participants spoke aspirationally with regards to their learning as opposed to descriptively. This demonstrates that context, experience and institutional affordances are important factors in transformative learning and therefore, transforming the learning. As Taylor and Cranton (2013) note, transformative learning is a deeply personal and context-bound journey, influenced by learners’ prior knowledge, emotional readiness, and social positioning.

Considering the trajectories through the ELT lens brings clarity to the language work that comes with CP. A key takeaway for students, that was valued by them, is that they are working with teachable linguistic resources for positioning ideas and the self, such as stance/hedging (e.g., epistemic modals, boosters, evidentials) and metadiscourse that manages audience engagement. This calls for designing speaking and writing tasks to build up the resources for stance/hedging and engagement explicitly through pedagogical activities like models, guided noticing, and criterion-referenced feedback, to establish CP as core outcomes of the instruction rather than an add-on (Hyland, 1998; Hyland, 2005). Especially in writing, metadiscourse has been a principled way of teaching how writers’ voices are brought into being, how arguments are organized, and how readers are guided in CP (Hyland, 2005). The Appraisal framework provides both instructor and learner with a common vocabulary suite for providing feedback and designing rubrics, i.e., Engagement (alignment with/against other voices), Attitude (evaluation), and Graduation (scaling claims). Making features visible made clear what was improved, and why, as the instruction aligned to CP (Martin and White, 2005). Several classroom episodes (e.g., interrogating coursebook texts, reframing tasks) lend themselves to Critical Language Awareness/Critical Discourse Analysis in ELT, as they provided regulatory systems for auditing stance, agency, and representation and coconstructing to redesign tasks (and broaden voices) to provide more equitable space-making; exactly the redesign/micro-teaching cycles seen here (Wallace, 1999; Cots). In addition, the participants’ ambivalence when considering materials reflects precisely what several studies of global ELT coursebooks have found: sanitized cultural representations and consumerist discourses that mask structural power, lending weight to the case for centering materials critique—and redesign—as a priority in the course (Gray, 2010).

In sum, this study provides compelling evidence that engagement with CP can foster holistic transformation in ELT graduate students. Through the deliberate cultivation of critical reflection, emotional awareness, and relational reciprocity, participants began to reimagine their teaching identities and practices in ways aligned with social justice and equity. These findings not only affirm the theoretical foundations of transformative learning and CP but also offer practical insights for curriculum design in teacher education programs. Embedding structured opportunities for reflection, dialogue, and experiential learning can nurture the conditions under which transformation flourishes—even within institutional constraints.

### Implications for practice

This study has demonstrated that engaging with CP in a structured teacher education context can facilitate meaningful transformation in ELT graduate students. Participants underwent cognitive, emotional, and relational shifts that enabled them to challenge assumptions about language teaching, reposition their identities, and imagine more equitable classroom practices. These findings affirm that transformative learning is not solely about acquiring new knowledge but becoming critically aware educators who are attuned to the sociopolitical dimensions of language teaching.

Regarding the implications of these findings, they present not only new insights into the power of CP in graduate-level ELT education, but also larger issues (even of multiple institutional interests) for classroom practice and educational policy in Türkiye. The participants obviously transformed their perceptions of themselves as a neutral knowledge transmitter to one who could be a reflective and socially engaged teacher, committed to providing inclusive, dialogic, and justice-oriented pedagogies. Furthermore, the data presented here potentially illustrates the need for ELT programs and curricula at the national level to more explicitly recognize the importance of critical pedagogies and frameworks, which recognize inequalities in societies, and provide educators with the resources and support to deal with the complexities and messiness of teaching for equity and transformation. Facilitating the implementation of critical pedagogies through institutional policy can provide recognition of and support to teachers’ emotional and intellectual engagement when discussing and enacting pedagogies of transformation. In order to do this, there is a need to move beyond technicist and exam-oriented approaches to teaching English and to foreground socially responsive, reflective, and dialogic pedagogies. Language teacher education programs should incorporate critical literacy tasks—such as analyzing textbook representations of gender, race, and culture—to help students interrogate hidden ideologies in instructional materials (Gray, 2010). Embedding such activities within reading and writing lessons can foster learners’ socio-political awareness while developing language skills. Moreover, structured reflective practices like weekly journaling, peer feedback cycles, and guided reflection prompts can support pre-service teachers in examining their beliefs and emotional responses to classroom challenges, thus enhancing critical consciousness (Farrell, 2015). Promoting inclusive material selection—such as using multimodal texts that reflect diverse voices and perspectives—can also validate learners’ identities and disrupt the cultural dominance often embedded in ELT curricula (Kubota, 2004). Additionally, implementing dialogic routines (e.g., critical roundtables, student-led discussions) encourages the co-construction of knowledge and models democratic classroom dynamics, a key principle of CP. To enable such practices, teacher education programs should also address institutional constraints and model pedagogical risk-taking, creating safe spaces where emerging educators feel supported in challenging normative teaching approaches.

Importantly, the study reveals that transformative learning is highly contextual. While many participants took intentional steps toward critical praxis, others hesitated due to structural constraints such as rigid curricula, exam pressures, or institutional hierarchies. This underscores the need for systemic support: teacher educators, school leaders, and policy-makers must create spaces that legitimize experimentation, dialogic learning, and pedagogical risk-taking. Without institutional backing, transformation risks remaining an individual aspiration rather than a collective educational shift.

These findings contribute to a deeper understanding of how engagement with CP can reshape ELT teacher identities, yet they also open important avenues for further inquiry. Future research could employ longitudinal designs to examine whether the cognitive and emotional shifts observed during teacher education translate into sustained classroom transformation. Comparative studies across different national or institutional contexts would also help illuminate how sociopolitical and policy environments shape the implementation of CP. In addition, mixed-method approaches linking teachers’ reflective narratives to concrete classroom practices and student learning outcomes could enrich the evidence base for equity-oriented ELT reforms. Furthermore, future studies could also explore the role of institutional affordances and constraints—such as curriculum flexibility, administrative support, or teacher autonomy—in enabling or hindering critical praxis. Identifying enabling conditions at the structural level would help inform teacher education programs and policy reforms that aim to foster equity-oriented pedagogical change.

## Limitations

While the study offers in-depth insights into the transformative learning experiences of graduate students enrolled in a single course on CP, its findings are context-bound and shaped by the specific institutional, cultural, and curricular dynamics of a foundation university in Türkiye. The small and homogeneous sample—comprised of a single cohort engaged in one course—limits the extent to which the results can be generalized to broader ELT populations or teacher education settings. As transferability in qualitative research relies on providing rich contextual detail (Lincoln and Guba, 1985), this study does not claim statistical generalizability but rather aims for analytical generalization by offering insights that may resonate with other educators and researchers in similar contexts. Future research could build on this study by exploring how CP is experienced across more diverse institutional types (e.g., state universities, in-service training programs), cultural settings, or levels of teaching experience to better understand the variables that influence transformative learning in ELT.

## Conclusion

This study reinforces the transformative potential of CP in ELT when supported by intentional design, reflective opportunities, and a safe, dialogic environment. When the course tasks focus on the language objectives and when the assessment criteria honor dialogic engagement alongside accuracy, range, and fluency, CP is not a competing agenda but another vehicle to achieve core ELT outcomes. Rather, in exam-oriented ecologies, that kind of alignment, combined with micro-teaching, criterion-referenced feedback, and iterative refinement, makes CP teachable and sustainable. Educators who undergo such transformation are better equipped not only to teach English, but to do so with an awareness of equity, justice, and the humanity of their students. As the participants’ journeys show, becoming a critically reflective and ethically committed teacher is not a one-time event, but a continuous act of learning, unlearning, and becoming.

## Data Availability

The original contributions presented in the study are included in the article/supplementary material, further inquiries can be directed to the corresponding author/s.

## Appendix

**Table A1:** Course plan.

Week	Focus	Language objectives	In-class activities
1	What is CP? Banking vs. problem-posing	Discuss CP using stance markers; frame reflective claims	Problem-posing demo; baseline reflective map
2	Power/Identity in ELT	Engagement with sources; hedging claims	Identity map; local constraints/affordances
3	Critical Language Awareness (CLA)	Metadiscourse: transitions and frame markers	Guided noticing of stance/representation
4	CDA mini-workshop	Evaluative lexis in analysis	Audit a coursebook/policy/media text
5	Materials and ınclusion	Attitude (Appraisal) in critique	Coursebook audit share-out; redesign proposals
6	From critique to design	Writing argument structure (claim–grounds–warrant)	Redesign tasks with explicit language aims and rubrics
7	Dialogic talk moves	Speaking stance & hedging; turn-taking	Talk-move rehearsal; feedback protocol
8	Micro-teaching #1	Oral metadiscourse; time management	Micro-teach + criterion-referenced feedback
9	High-stakes exams and washback	Test-genre register; planning under time	Exam wrapper; low-stakes rehearsal
10	Assessment for Learning (feedback)	Feedback language; Graduation	Build analytic rubrics; redo cycles
11	CP in Speaking	Stance & hedging in debate	Debate task design + rehearsal
12	CP in Writing	Evaluative lexis & argument structure	Op-ed design; source integration
13	Ethics, care, teacher emotion	Reflective Engagement with counter-voices	Case clinic; boundary-setting
14	Micro-teaching #2 and synthesis	Integrated stance/argument; reflective voice	Micro-teach #2; action plans
